# Supplementary material for: In-Silico and In-Vitro Analysis of the Novel Hybrid Comprehensive Stage II Operation for Single Ventricle Circulation
Source: Bioengineering (Basel). 2023 Jan 19;10(2):135. doi: 10.3390/bioengineering10020135 (PMC9952694; doi:10.3390/bioengineering10020135)
Supplement: Supplementary file 1 [file bioengineering-10-00135-s001.zip › bioengineering-1910710-supplementary.pdf]

Supplementary Material

In-silico and In-vitro Analysis of the Novel Hybrid Comprehensive Stage II Operation for Single Ventricle Circulation

Arka Das[1*], Marwan Hameed[2], Ray Prather[1,3,4], Michael Farias[4,5], Eduardo Divo[1], Alain Kassab[3], David Nykanen[4,5], and William DeCampli[4,5]

[1] Department of Mechanical Engineering, Embry-Riddle Aeronautical University; Daytona Beach, FL 32114, USA
[2] Department of Mechanical Engineering, American University of Bahrain, Bahrain
[3] Department of Mechanical and Aerospace Engineering, University of Central Florida; Orlando, FL 32816, USA
[4] The Heart Center at Orlando Health Arnold Palmer Hospital for Children, Orlando, FL 32806, USA
[5] College of Medicine, University of Central Florida, Orlando, FL 32816, USA
* Correspondence: dasa@erau.edu; Tel.: +1-386-241-1457

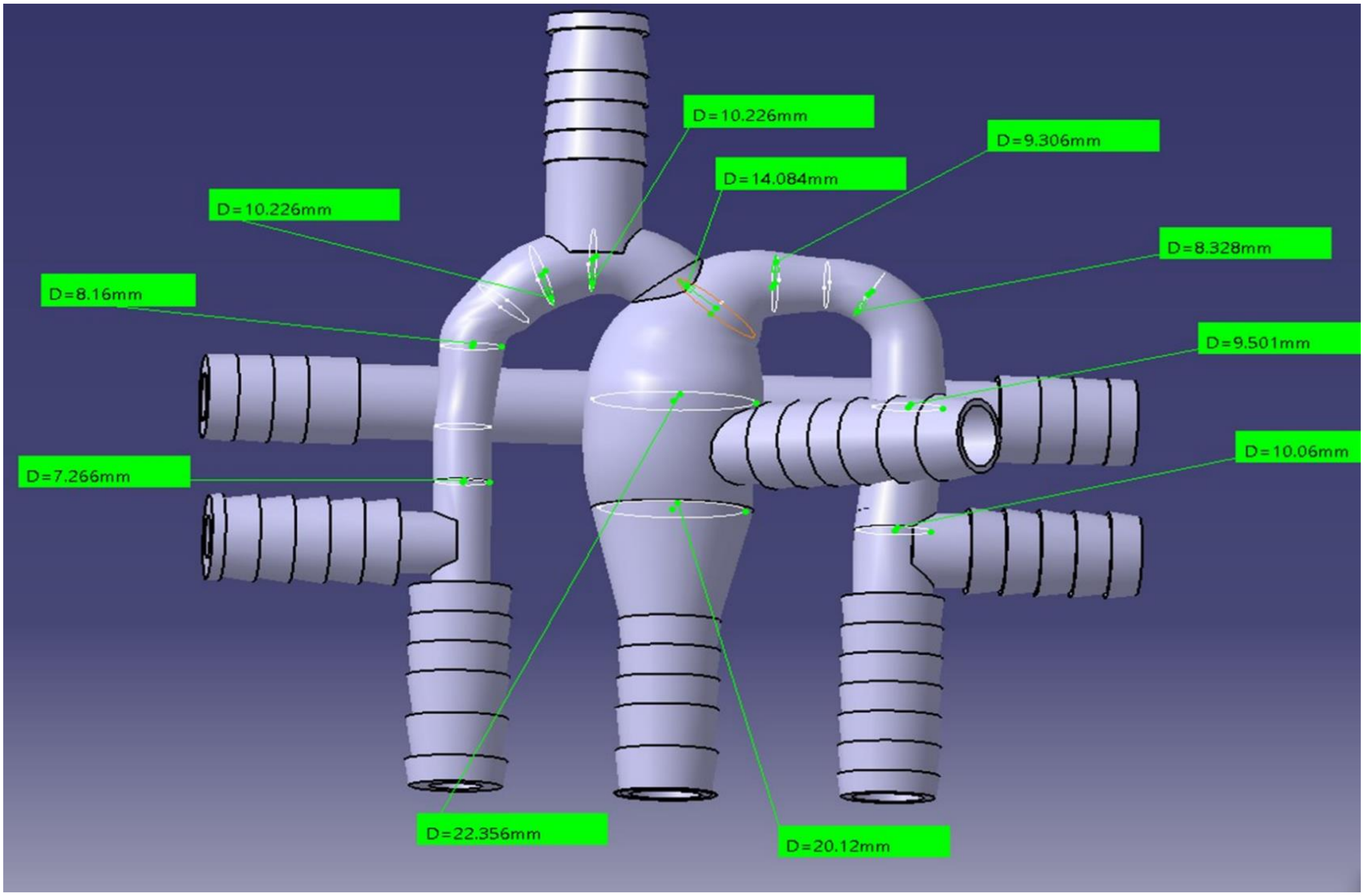

Figure S1. - Development of 3D CAD model of HCSII centerpiece from deidentified angiographic images for performing in-vitro studies. Dimension of the 3D phantom with the labeled diameters (in mm) of labeled branches using CATIA v5.

Table S1. Statistical analysis using One way ANOVA for in-vitro PRT study of patient 1

Source of Variation	S.S.	df	M.S.	F	P-value	F crit
Between Groups	0.715123	4	0.178781	0.747943	0.566046	2.641465
Within Groups	8.366052	35	0.23903			
Total	9.081175	39				

Table S2. Statistical analysis using One way ANOVA for in-vitro PRT study of patient 2

Source of Variation	S.S.	df	M.S.	F	P-value	F crit
Between Groups	0.000556	2	0.000278	0.022839	0.977448	3.554557
Within Groups	0.218958	18	0.012164			
Total	0.219513	20				

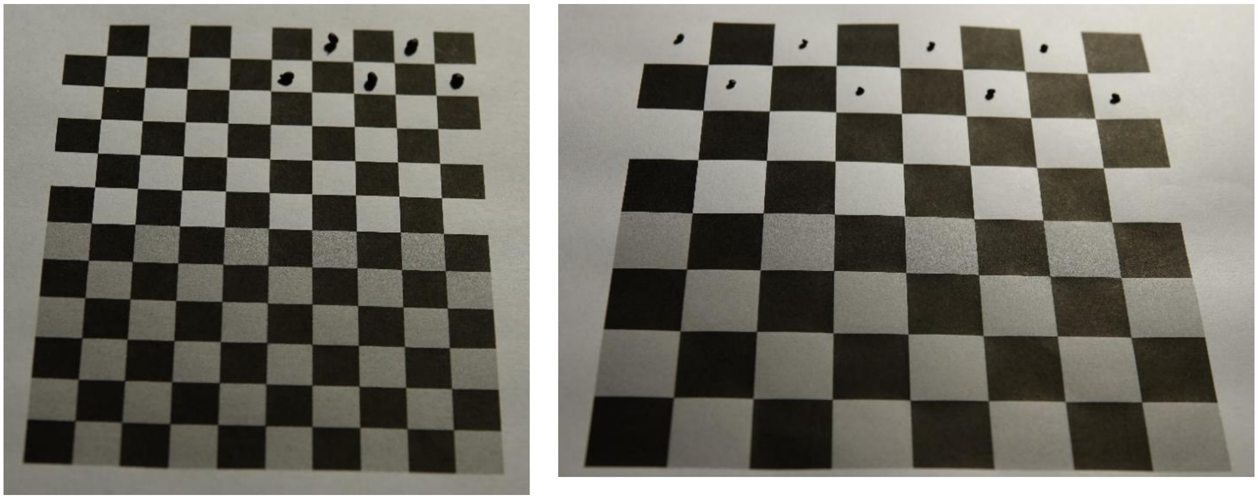

(**a**) (**b**)

Figure S2. The binary checkerboard patterns used for HS camera calibration purposes in in-vitro PRT study containing (a) 5 x 6 (Binary) and (b) 8 x 8 (binary) patterns.

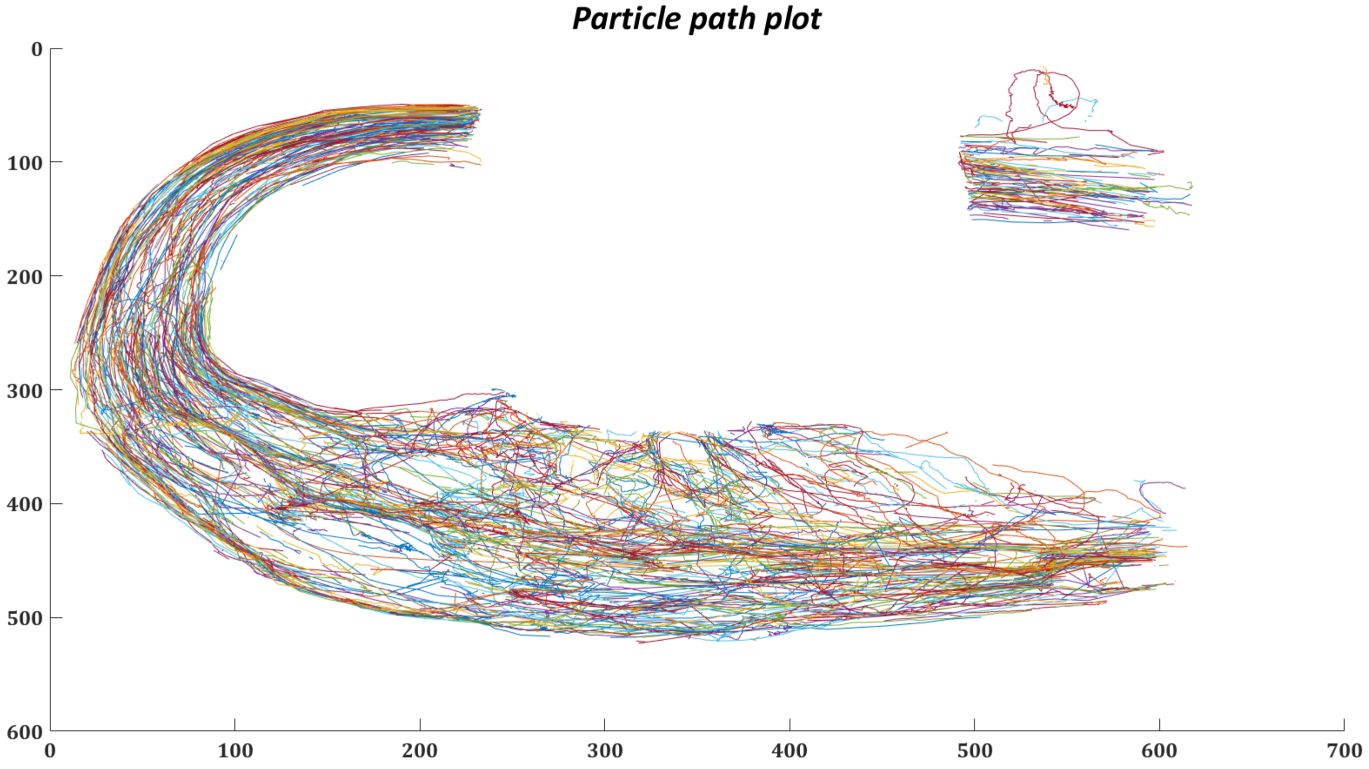

Figure S3. – Trajectory plot of tracked particles in the MPA conduit for patient 1 obtained from experimental trial 3.

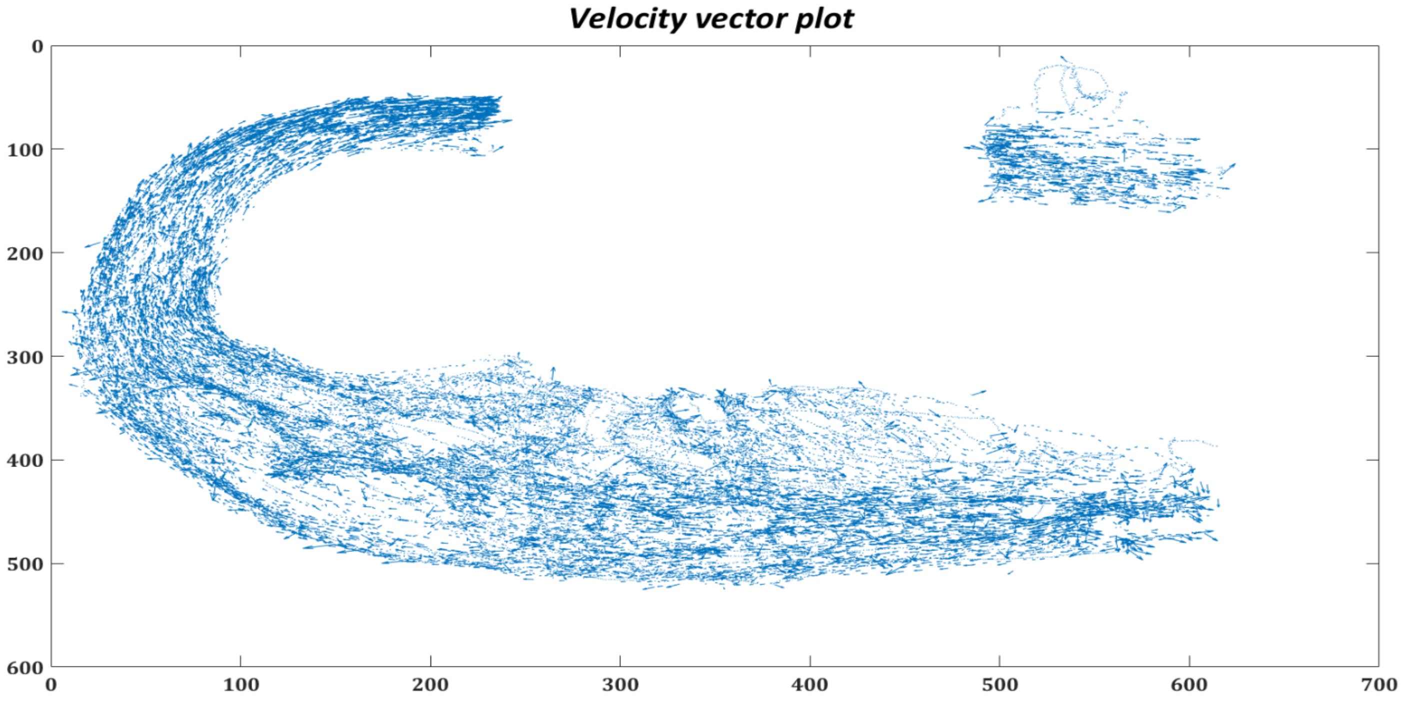

Figure S4. – Velocity vector plot of the tracked particles in the MPA conduit for patient 1. obtained from experimental trial 3

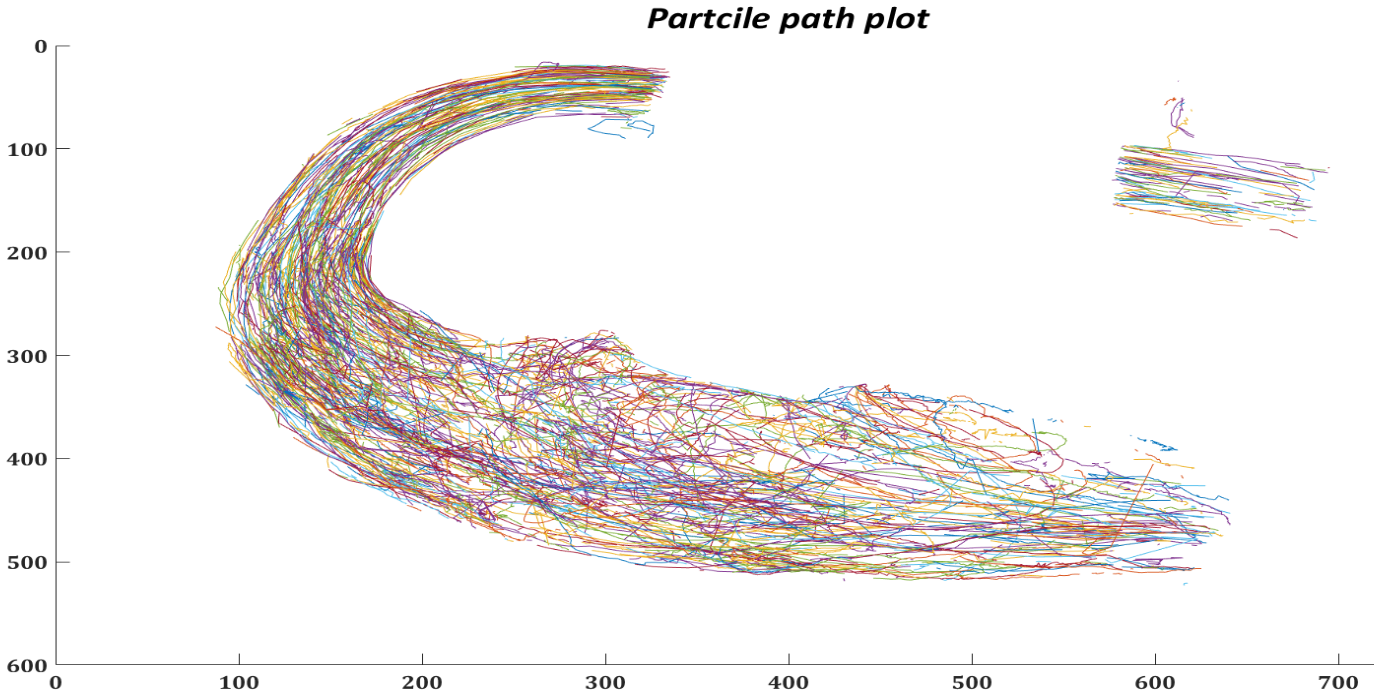

Figure S5. – The trajectory plot of tracked particles in the MPA conduit for patient 1 in experimental trial 4.

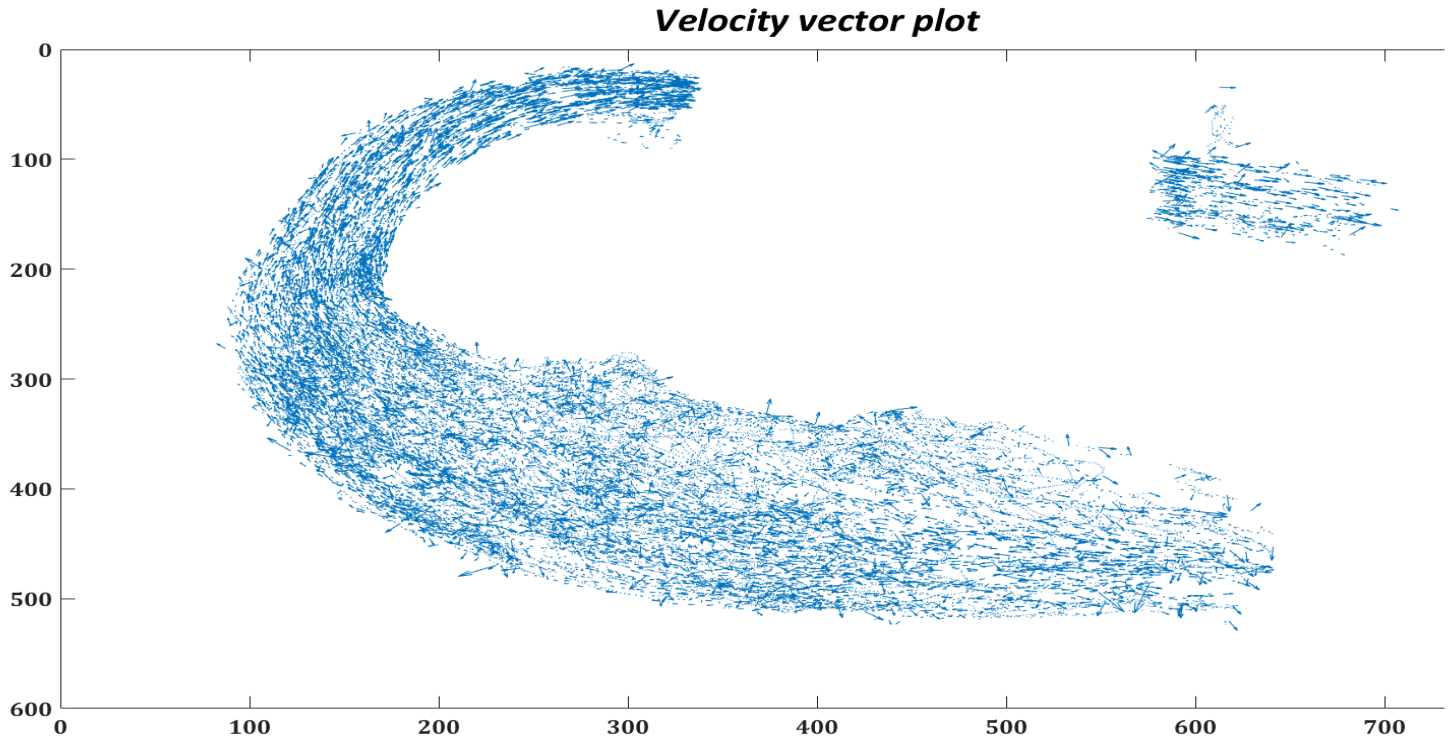

Figure S6. – Velocity vector plot of the tracked particles in the MPA conduit for patient 1 obtained from experimental trial 4.

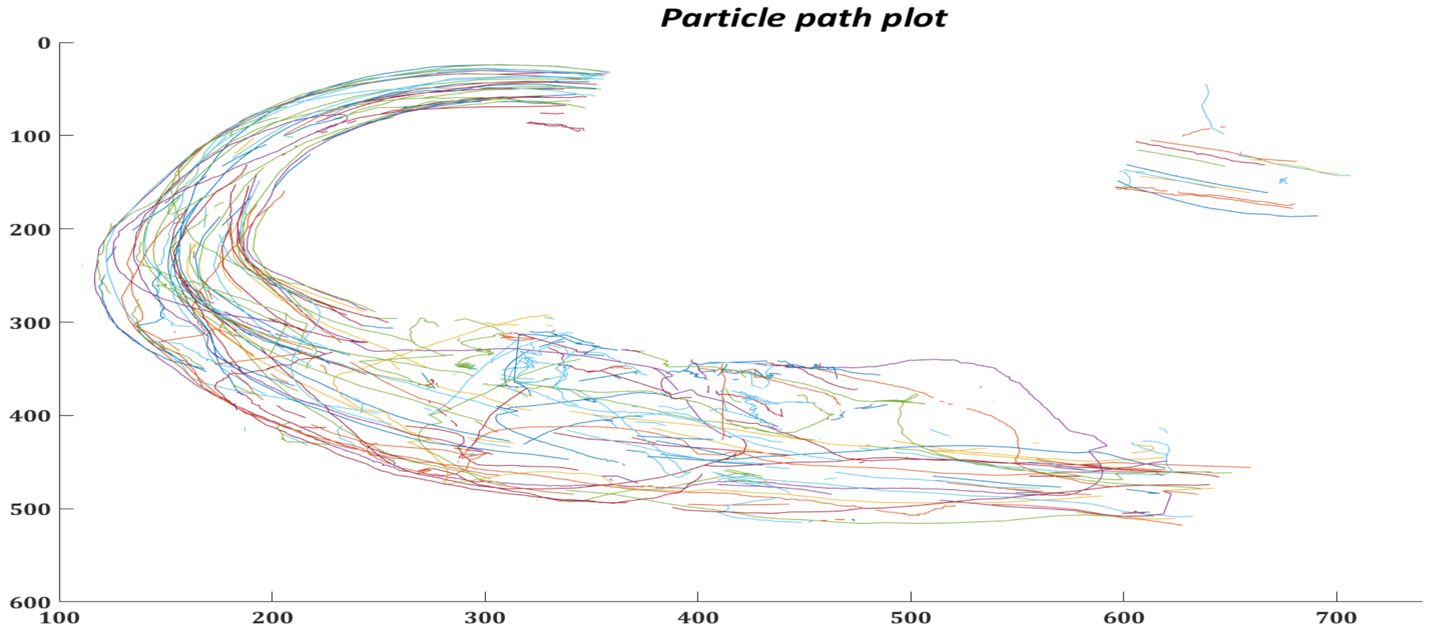

Figure S7. – The trajectory plot of tracked particles in the MPA conduit for patient 1 in experimental trial 5.

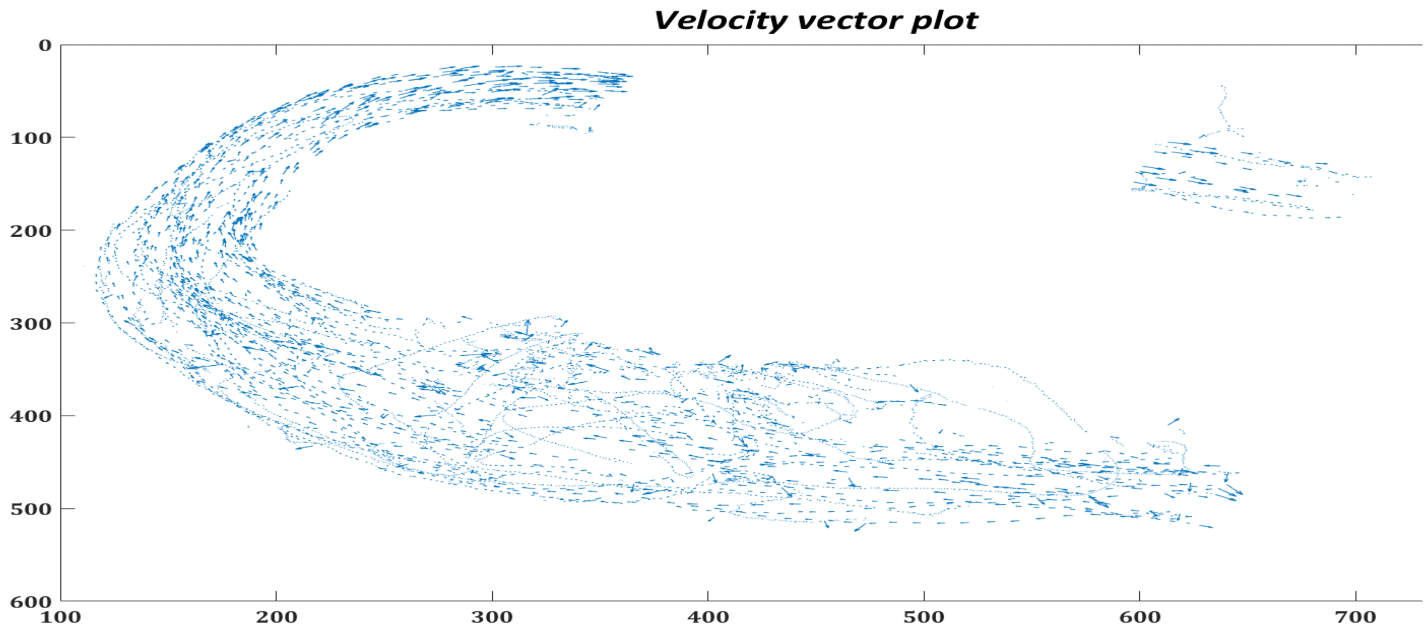

Figure S8. – Velocity vector plot of the tracked particles in the MPA conduit for patient 1 obtained from experimental trial 5.

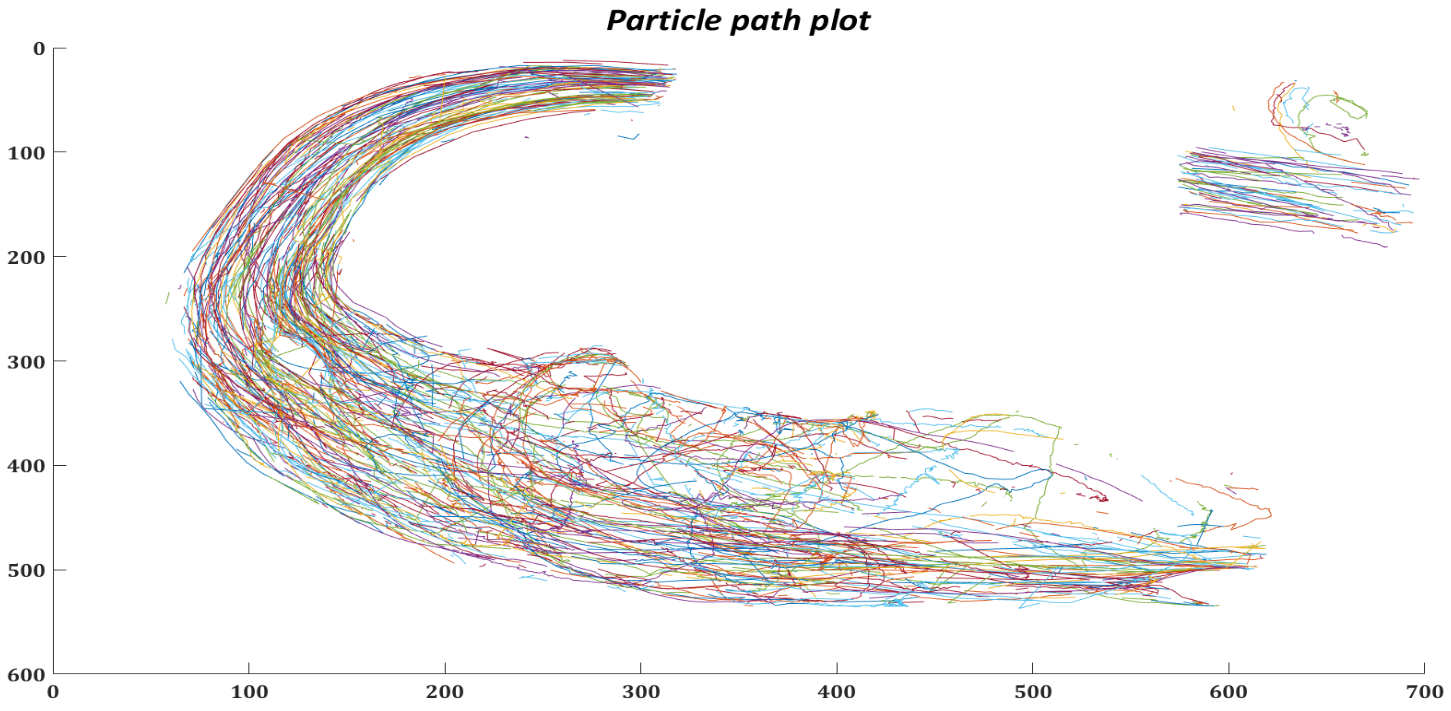

Figure S9. – The trajectory plot of tracked particles in the MPA conduit for patient 1 in experimental trial 6.

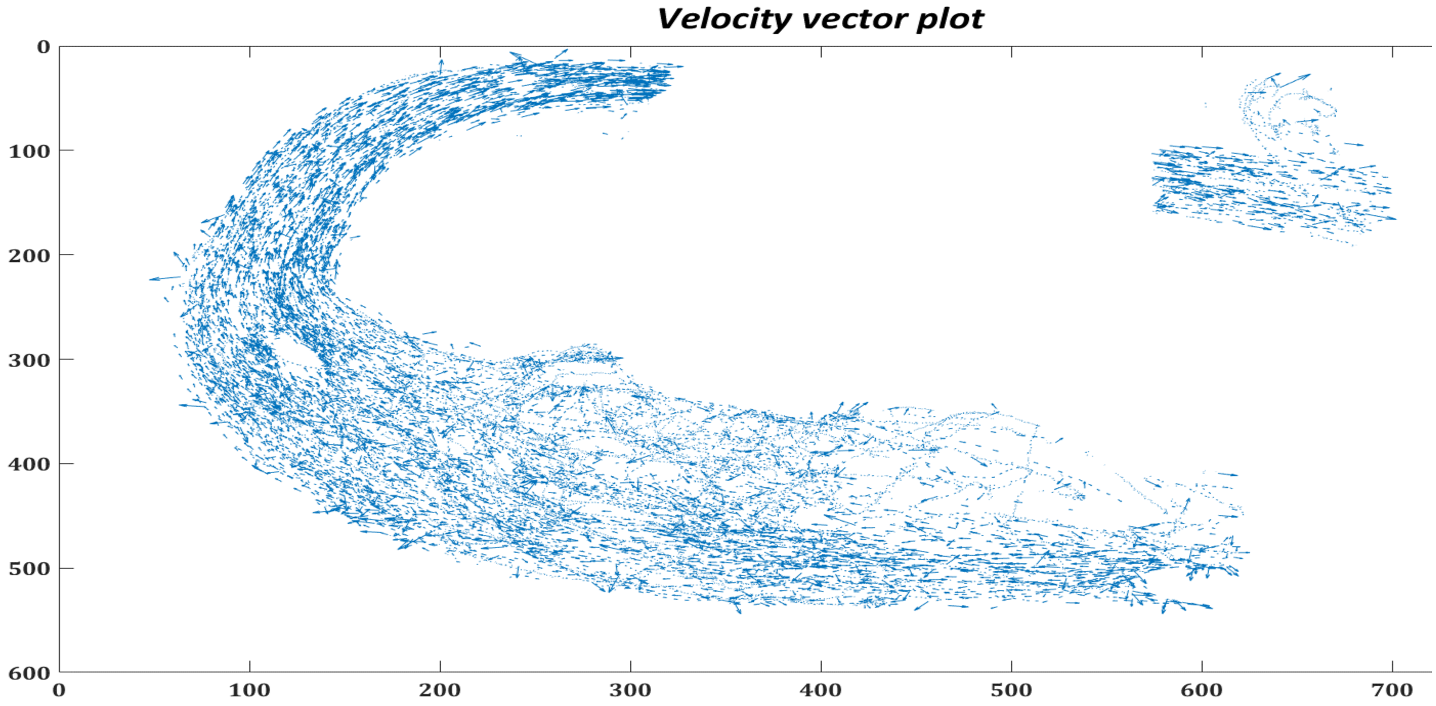

Figure S10. – Velocity vector plot of the tracked particles in the MPA conduit for patient 1 obtained from experimental trial 6.

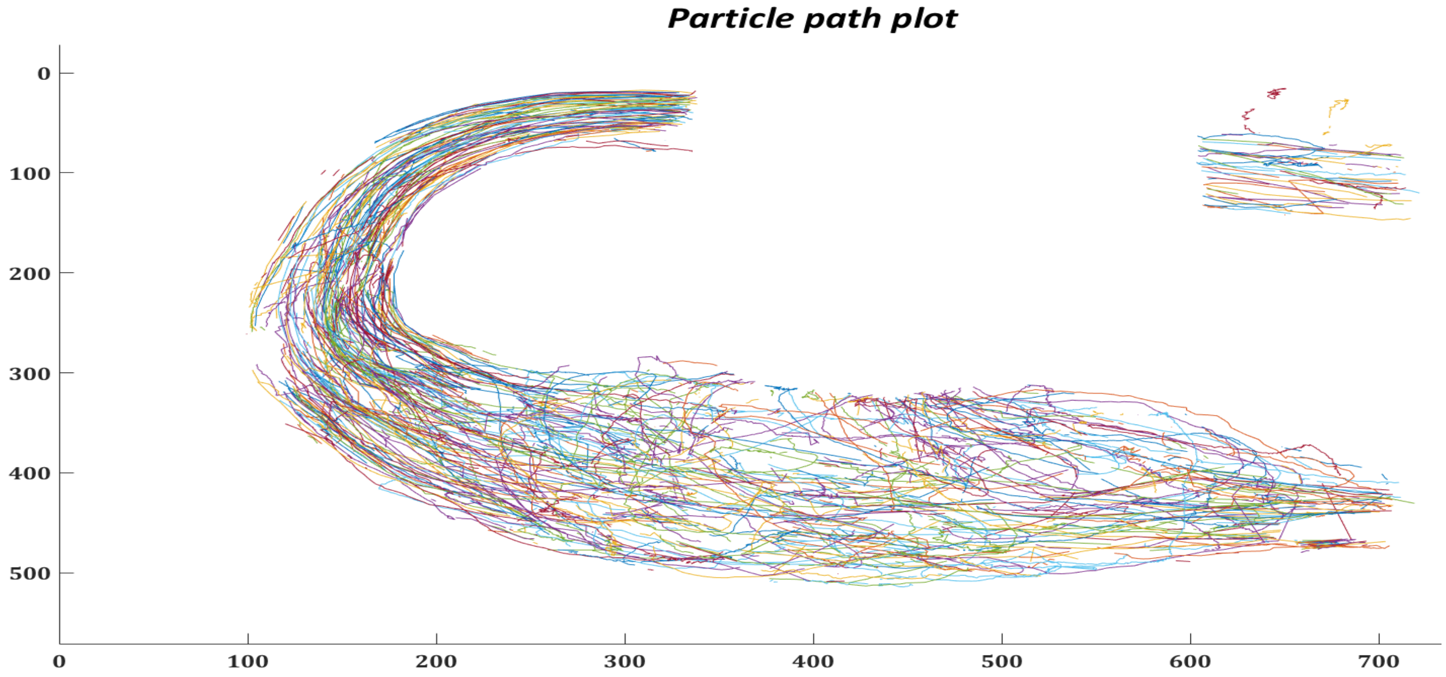

Figure S11. – The trajectory plot of tracked particles in the MPA conduit for patient 2 obtained from experimental trial 1.

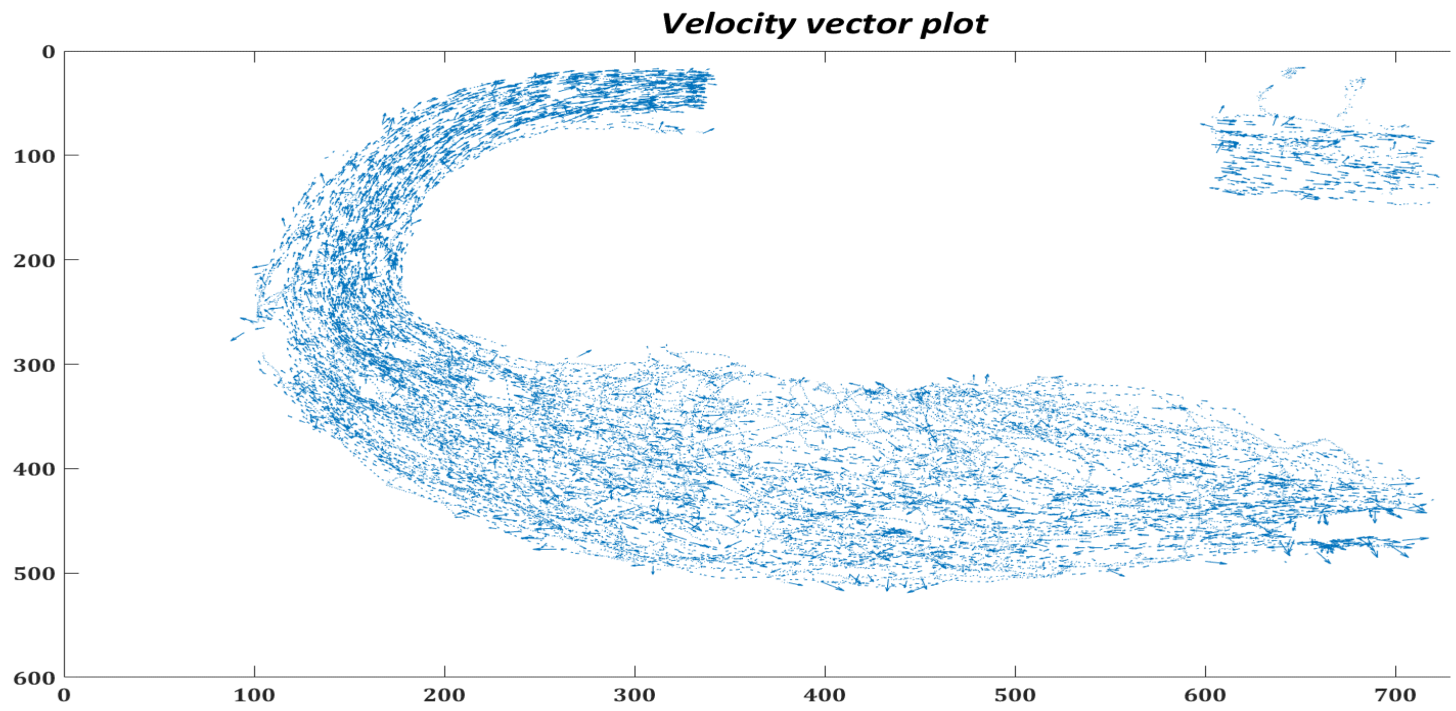

Figure S12. – Velocity vector plot of the tracked particles in the MPA conduit for patient 2 obtained from experimental trial 1.
